# Actin cable formation and epidermis–dermis positional relationship during complete skin regeneration

**DOI:** 10.1038/s41598-022-18175-y

**Published:** 2022-09-23

**Authors:** Kento Takaya, Keisuke Okabe, Ayaka Ishigami, Yuka Imbe, Hideko Kanazawa, Shigeki Sakai, Noriko Aramaki-Hattori, Kazuo Kishi

**Affiliations:** 1 Department of Plastic and Reconstructive Surgery, School of Medicine, Keio University, 35 Shinanomachi, Shinjukuku, Tokyo, 160-8582 Japan; 2 Faculty of Pharmacy, Keio University, Shiba, Minatoku, Tokyo, Japan

**Keywords:** Cell biology, Structural biology, Diseases, Molecular medicine

## Abstract

Up to a certain developmental stage, a fetus can completely regenerate wounds in the skin. To clarify the mechanism of fetal skin regeneration, identifying when the skin switches from fetal-type wound regeneration to adult-type wound repair is necessary. We hypothesized that this switch occurs at several time points and that complete skin regeneration requires epidermal–dermal interactions and the formation of actin cables. We compared normal skin and wound morphology at each developmental stage. We examined two parameters: epidermal texture and dermal structure. We found that the three-dimensional structure of the skin was completely regenerated in full-thickness skin incisions made before embryonic day (E) 13. However, the skin texture did not regenerate in wounds made after E14. We also found that the dermal structure regenerates up to E16, but wounds created after E17 heal as scars with dermal fibrosis. By controlling the activity of AMP-activated protein kinase and altering actin cable formation, we could regulate scar formation in utero. These findings may contribute to therapies that allow complete skin regeneration without scarring.

## Introduction

Regeneration is among the most important topics in modern medicine. In lower animals, regeneration is observed in tailed amphibians and planaria^1–3^. Conversely, in highly-developed animals such as mammals, only a few organs, such as the liver and fingertips, are capable of regeneration^4,5^. Moreover, when a skin wound occurs in an adult animal, the structure never fully regenerates during healing, leaving a scar^6^. Currently, there is no clinical method to completely restore scar tissue to its original state. In contrast, fetal skin wounds can regenerate up to a certain developmental stage without leaving scars^7–10^. Fetal regeneration has been demonstrated in various tissues and may serve as a model for tissue regeneration. There has been great interest in determining at which stage during late fetal development does the skin loses the ability to completely regenerate and transitions to a scarring phenotype similar to that of adult animals. Previous reports describe a gradual transition of fetal wound regeneration to adult wound healing and scarring, but this "gradual transition" makes it difficult to analyze the mechanism of fetal regeneration^11,12^.

Scar tissue is characterized by (1) dermal fibrosis, (2) loss of skin texture, (3) loss of skin appendages, and (4) changes in color tone^13,14^. Therefore, to evaluate scar morphology (excluding color tone), it is necessary to observe regeneration in two ways: skin texture and dermal structure. Previous studies on wound healing using mouse fetuses showed that wounds created between embryonic day 15 (E15) and E16 regenerate; however, this has been evaluated only by the observation of histological parameters indicative of the presence of dermal fibrosis^15–18^.

In our previous study, skin texture did not regenerate after wounding in E15 mice. Thus, wound healing at this developmental stage is not considered complete regeneration, as visible marks remain in the area after healing^19^. Therefore, establishing a fetal mouse model for complete skin regeneration is critical. However, creating wounds during the early embryonic stages is technically difficult. Using our recently-developed microsurgical method, we were able to elucidate the timing of the transition between the presence and absence of skin texture regeneration after wound creation in early embryonic mouse fetuses^10,20^. In this study, we showed the detailed morphological changes before and after the transition from regeneration to non-regeneration.

In addition, a relationship between skin regeneration and actin cable formation at the epithelial margin was proposed by Martin et al.^21^. Actin cable formation refers to actin filament cables that extend across cells at the tip of the healing embryonic epithelium and appear to form a ring around the wound. These cables are hypothesized to function as a contractile "drawstring" that facilitates wound closure^22^. In this study, we clarified the relationship between the presence of actin cable formation and changes in skin regeneration parameters before and after the transition. In adult mammalian wounds, keratinocytes migrate over the exposed connective tissue using foliar pseudopodia to close the wound^23^. However, early embryonic fetal wounds in whole embryo cultures do not show lobular pseudopodia, and the epidermis appears to have thread-like actin cables running continuously over most of the wound margin^24^. The purse-like contraction of these actin cables is critical to wound healing in fetal tissue but is not observed in fetal wounds in vivo. AMP-activated protein kinase (AMPK) and its substrate, PDZ and LIM domain protein 5 (PDLIM5), regulate cell migration by controlling microtubule and actin filament behavior^24^.

We hypothesized that the mechanism of skin regeneration, which has been described as gradual, has a tipping point for each parameter, and that this may be connected to the formation of actin cables.

This study aimed to analyze the conversion point in mouse fetal wound healing between a regenerative to a non-regenerative phenotype. We also clarified the role of actin cables in mouse wounds before and after this transition. Our results revealed the point at which fetal wound regeneration transforms into adult scar wound healing. Next, we investigated how modulating actin cable formation affects scarring. These results provide evidence for the development of therapies for complete skin structure regeneration.

## Results

### Transition of skin texture and dermal structure in fetal mice

**Figure 1:**
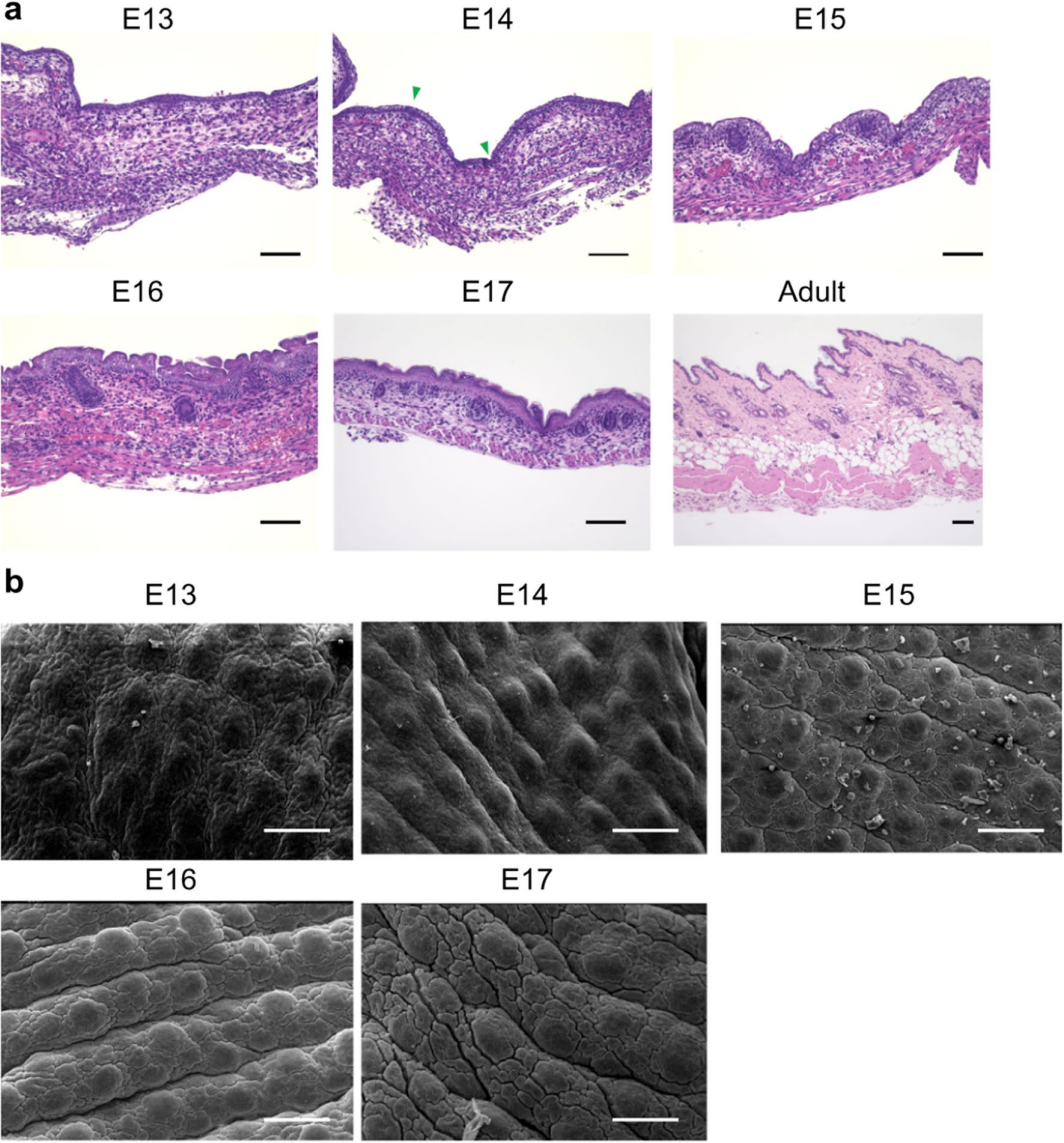
Skin structure in mice fetuses. (**a**) H&E staining, Scale bar = 100 µm. (**b**) SEM image, Scale bar = 200 µm. Skin appendages were not observed in E13 wounds, but epidermal skin texture was present. At later developmental stages, the skin texture was deep and clearly formed. In E14 wounds, placodes and dermal condensation were observed, indicating the onset of hair follicle formation. Hair follicles began to appear from E15. From E16 onward, the tissue structure was equivalent to that of adult mice. Green arrow: cohesive part of the dermis (placode formation).

No hair follicles or appendages were observed at E13 in normal skin. However, the epidermal surface at E13 was not smooth, i.e., the skin texture was already present. As the fetus developed, the skin texture became more uneven. From E14, placode and dermal condensation were observed, indicating the onset of hair follicle formation. Hair follicles began to appear from E15. From E16 onward, the tissue structure was comparable to that of the adult mice (Fig. 1a,b).

**Figure 2:**
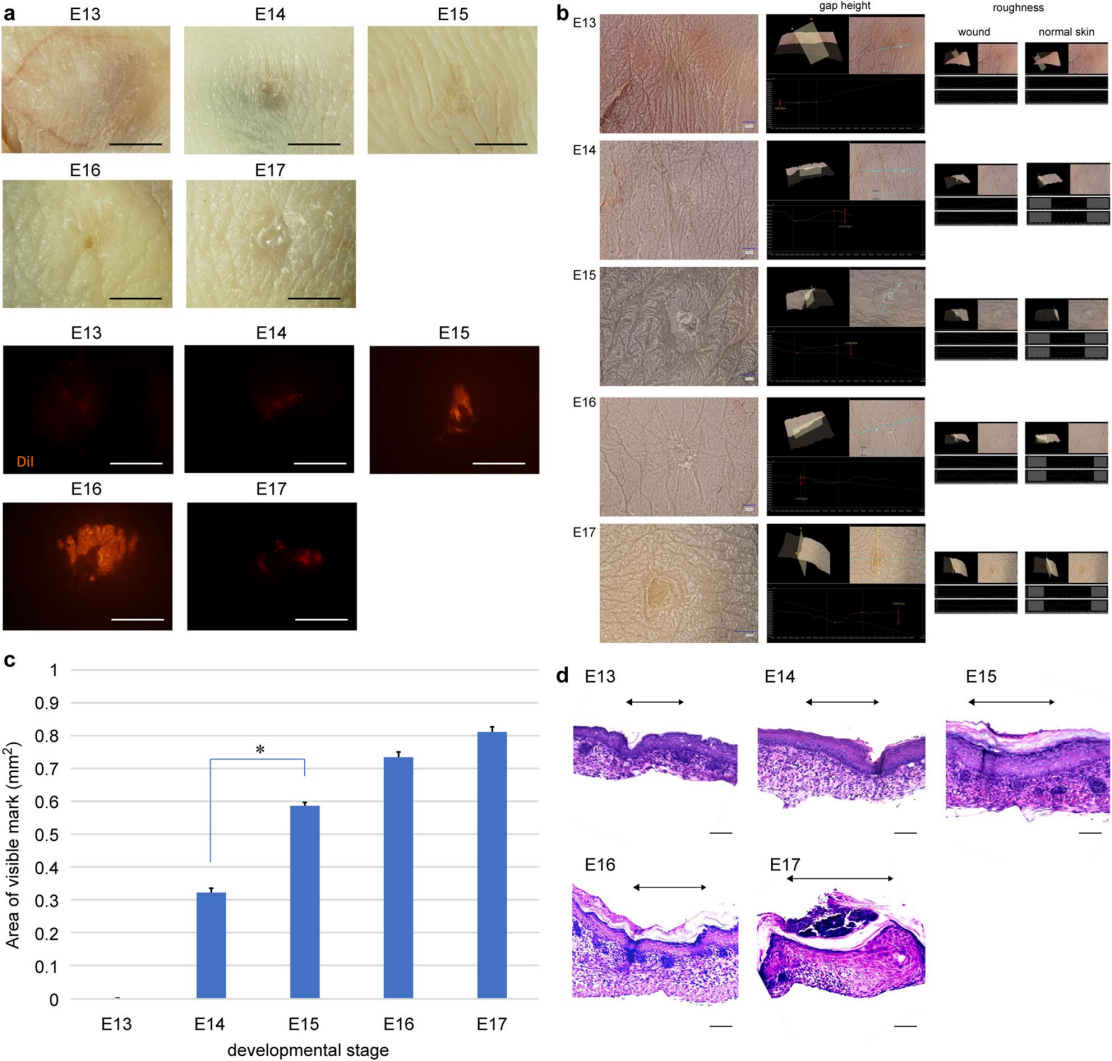

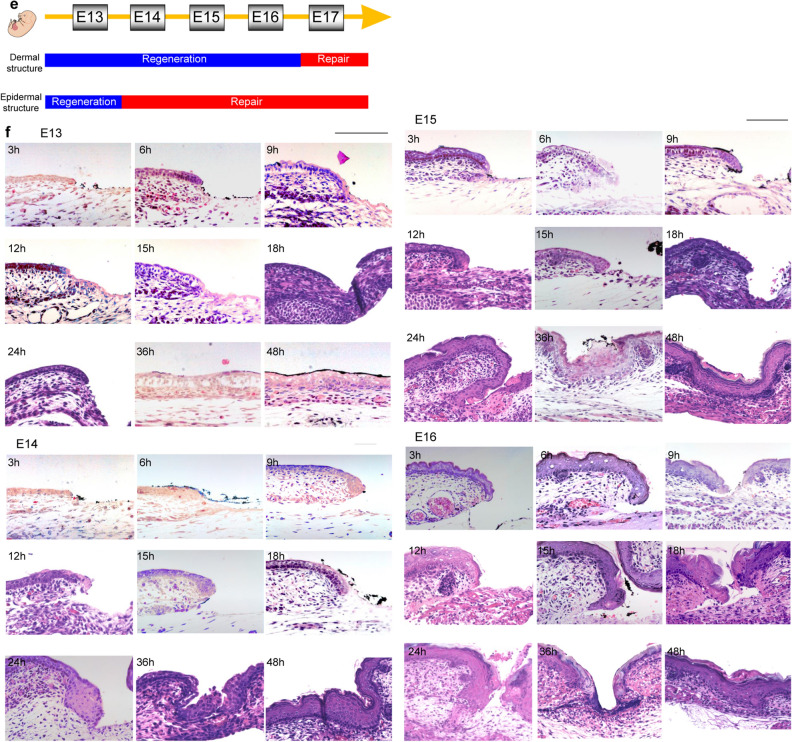

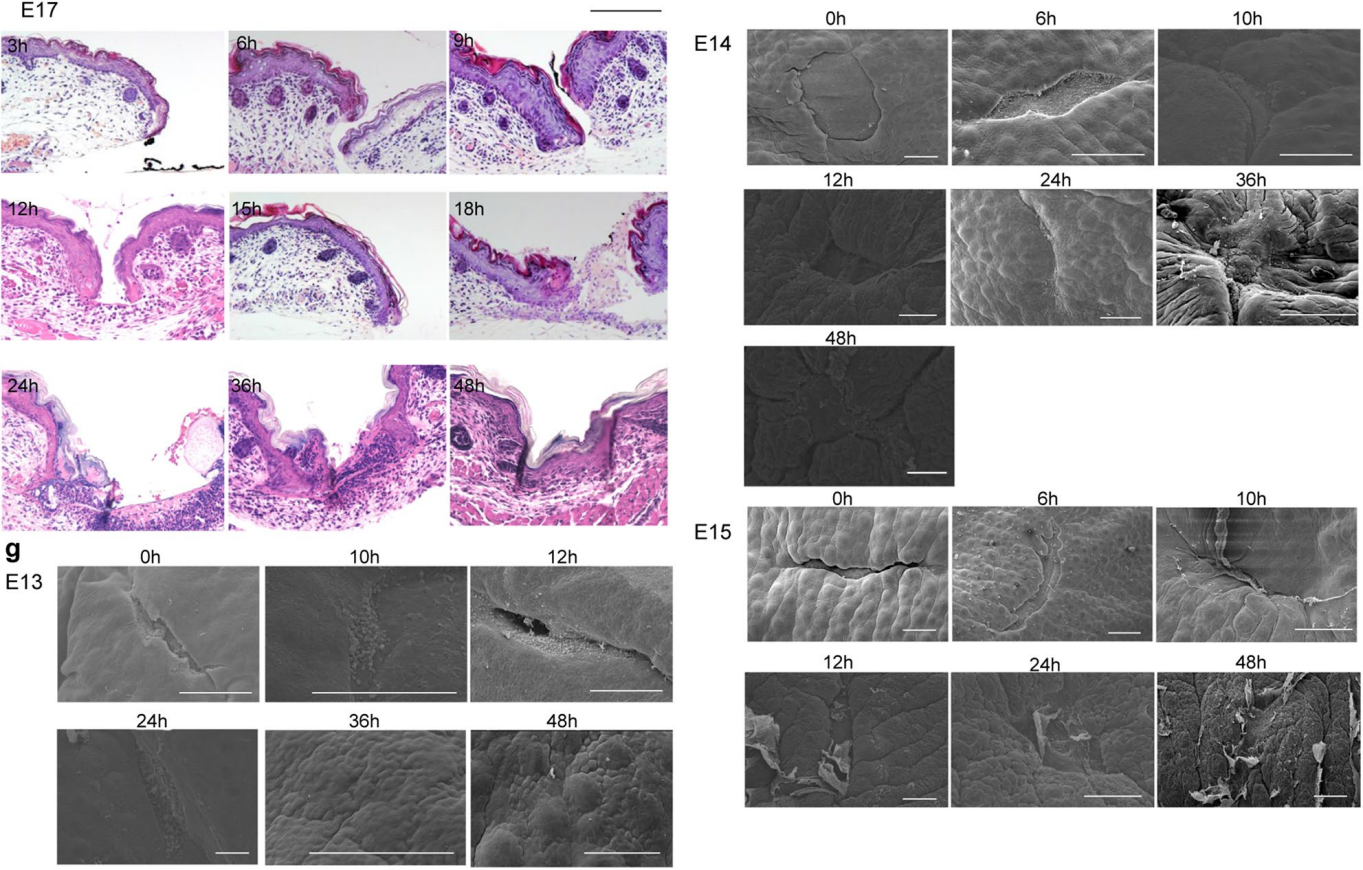
Wound healing in mouse fetuses. (**a**) Gross photograph at 72 h after wounding showing complete skin regeneration without scarring in E13 wounds. Visible marks were observed in wounds made at E14 or later. All wounds showed subcutaneous DiI uptake, which labeled the wound site. Scale bar = 1 mm. (**b**) Representative image obtained 72 h after wounding. The skin texture was regenerated, and the wound was almost free of depression compared to the surrounding normal skin in E13 wounds, but a visible mark and depressions in the surrounding skin were observed in E14 wounds. In addition, inside the visible mark in E14 wounds, the roughness was reduced compared to that of normal skin. (**c**) Comparison of the wound 72 h after wounding. n = 5 for each developmental stage. (**d**) H&E staining of the wound after 72 h showing healing of the dermis and other structures up to E16, but the dermis was replaced by thick scar tissue at E17. Scale bar = 100 µm. (**e**) Timing of the transition between epidermal and dermal regeneration during mouse development. (**g**) Regeneration of the epidermis and dermis over time, focusing on the wound margin. H&E images. In E13 wounds, the dermis moved into the skin defect earlier than the epidermis. In wounds made at E14 or later, the epidermis moved into the wound earlier than the dermis, and the leading edge contacted the fascia. In E16 wounds, the dermis regenerated, but in E17 wounds, cells derived from the fascia accumulated in the wound, forming scar tissue. Scale bar = 100 µm. (**g**) SEM images of wound healing at E13, E14, and E15. Scale bar = 200 µm.

We used microsurgical scissors with one serrated blade to create wounds of uniform depth that reached the loose fascia beneath the panniculus carnosus muscle. The wounds did not reach the deep muscle layer at any developmental stage from E13 to E17. The uniform wound depth is due to the very loose and mobile fascial layer between the panniculus carnosus and the deep muscle. All fetal wounds re-epithelialized after 48 h and the scars were assessed 72 h after wounding. When the wound surfaces were examined at 72 h post-wounding, wounds made at E13 had completely regenerated epidermal texture, whereas wounds made from E14 to E17 did not regenerate the skin texture and left a visible mark. In the microscopy analysis, the texture pattern regenerated at E13, and the wound was almost free of depressions compared with the surrounding normal skin. In contrast, at E14 and later, a visible mark remained and depressions in the surrounding skin were observed. (Fig. 2a,b). When the wounds created with a 1-mm diameter skin biopsy punch were assessed after 72 h, the E14 wounds were significantly smaller than those made after E15 (P = 0.0003) (Fig. 2c). These results suggest that skin healing at E14 proceeded in a regenerative manner. Histological observation showed that the dermal structure regenerated in wounds made at E13–E15, whereas it did not regenerate and fibrotic scar tissue with condensed collagen remained in the dermis in wounds made at E16–E17 (Fig. 2d). These results indicate that the transition from complete skin and dermal structure regeneration to no skin regeneration occurs at E13 and E14 and that the transition from when the dermal structure regenerates to when fibrosis occurs is at E16 and E17 (Fig. 2e).

A more detailed observation of the wound healing process over time showed a difference in the positional relationship between the epidermis and dermis at the wound edge between E13 and E14. In wound healing at E13, the dermis moved to and covered the skin defect earlier than the epidermis, and the wound closed without disturbing the positional relationship between the epidermis and dermis. In contrast, in wound healing from E14 to adulthood, the epidermis moved into the wound faster than the dermis, resulting in wound closure after the edge of the epidermis touched the fascia (Fig. 2f). This morphological change suggests that interactions between the epidermis, dermis, and fascia are involved in the regenerating skin texture. In wound healing up to E16, after re-epithelialization was complete and the epidermis and fascia made contact, the dermis moved through the layer underneath the re-epithelialized epidermis toward the wound. This dermal layer regenerated a normal structure 72 h after wounding. In E17 wounds 72 h after wound creation, the dermal cells did not migrate under the re-epithelialized epidermis, and the cells of the fascia within the wound eventually formed scar tissue (Fig. 2f).

### Epidermal–dermal positional relationship plays a key role in skin texture regeneration

**Figure 3:**
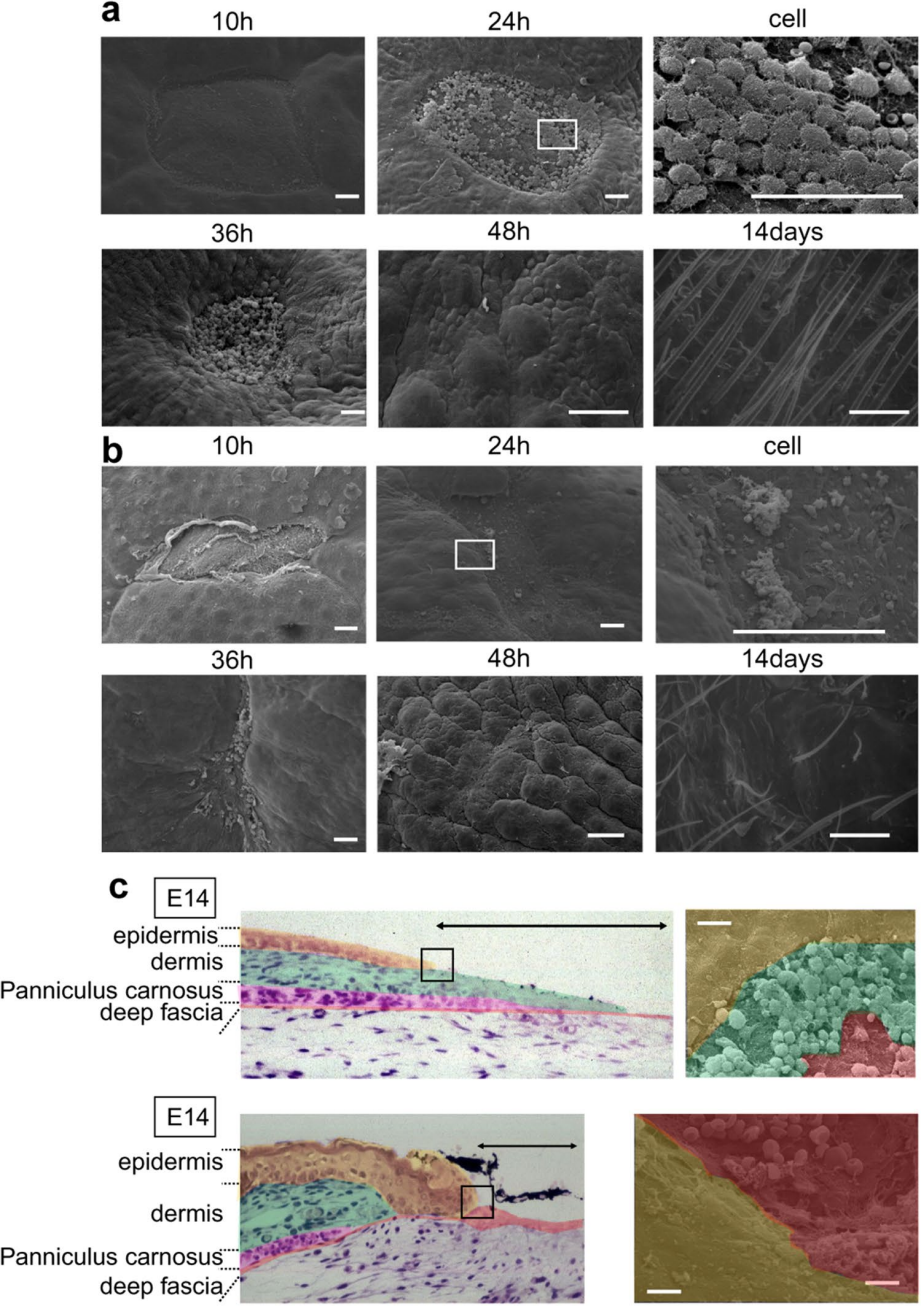
SEM images of fetal wound healing. (**a**) E13 wound, (**b**) E14 wound. Scale bar = 100 µm. (**c**) Difference in epidermal–dermal interactions at the wound margin between E13 and E14. In E13 wounds, the epidermis and dermis remained in position, the wound closed, and epidermal–dermal interactions were always observed. In E14 wounds, epidermal–fascial interactions were observed, even if only temporarily. Scale bar = 20 µm.

To observe the differences in epidermal–dermal interactions between E13 and later developmental stages, we performed a three-dimensional observation of the wound via scanning electron microscopy (SEM). In E13 fetal wounds, the wound spontaneously detached after wound creation, and the fascial layer beneath the panniculus carnosus contained many spindle-shaped cells in a small amount of fibrous tissue. The E13 dermis, however, was dominated by spherical cells. Ten hours after wounding, the spherical cells that emerged from the edges of the wound aggregated. The base of the wound and the fascial layer were covered with flattened cells. Twenty-four hours after wounding, the wound became smaller, and most of the wound site was covered with spherical cells. At this point, the spherical cells grew many protrusions and were connected to each other. Thirty-six hours after wounding, the wound was completely covered with spherical cells. The epidermis above the spherical cells completely closed the wound after 48 h. At the same time, the skin texture had completely regenerated (Fig. 3a).

Transmission electron microscopy (TEM) observation 24 h after wounding at E13 showed that cells in the wounded area had chromatin-rich nuclei, little cytoplasm, and morphology similar to dermal fibroblasts (Supplementary Fig. 1). In addition, these cells were connected to the dermal layer, but not to the fascial layer. These morphological observations showed that starting at E13, the epidermis was located above the dermis during skin regeneration.

During wound healing in E14 fetuses, no spherical cells were present at the wound edges 10 h after wounding. The epidermis grew mainly on flat fascial cells, and the epidermis and fascia were in contact 24 h after wound creation. The epidermal pattern did not regenerate, and a visible mark remained in the wound center (Fig. 3b). SEM and histological observations confirmed that in wound healing at E13, epithelialization is completed while the epidermal–dermal positional relationship is always maintained. However, in wound healing at E14, a different positional relationship is observed where the epidermis temporarily overtakes the dermis and contacts the fascia (Fig. 3c).

### Transition from wound healing to cell migration by actin cable formation

**Figure 4:**
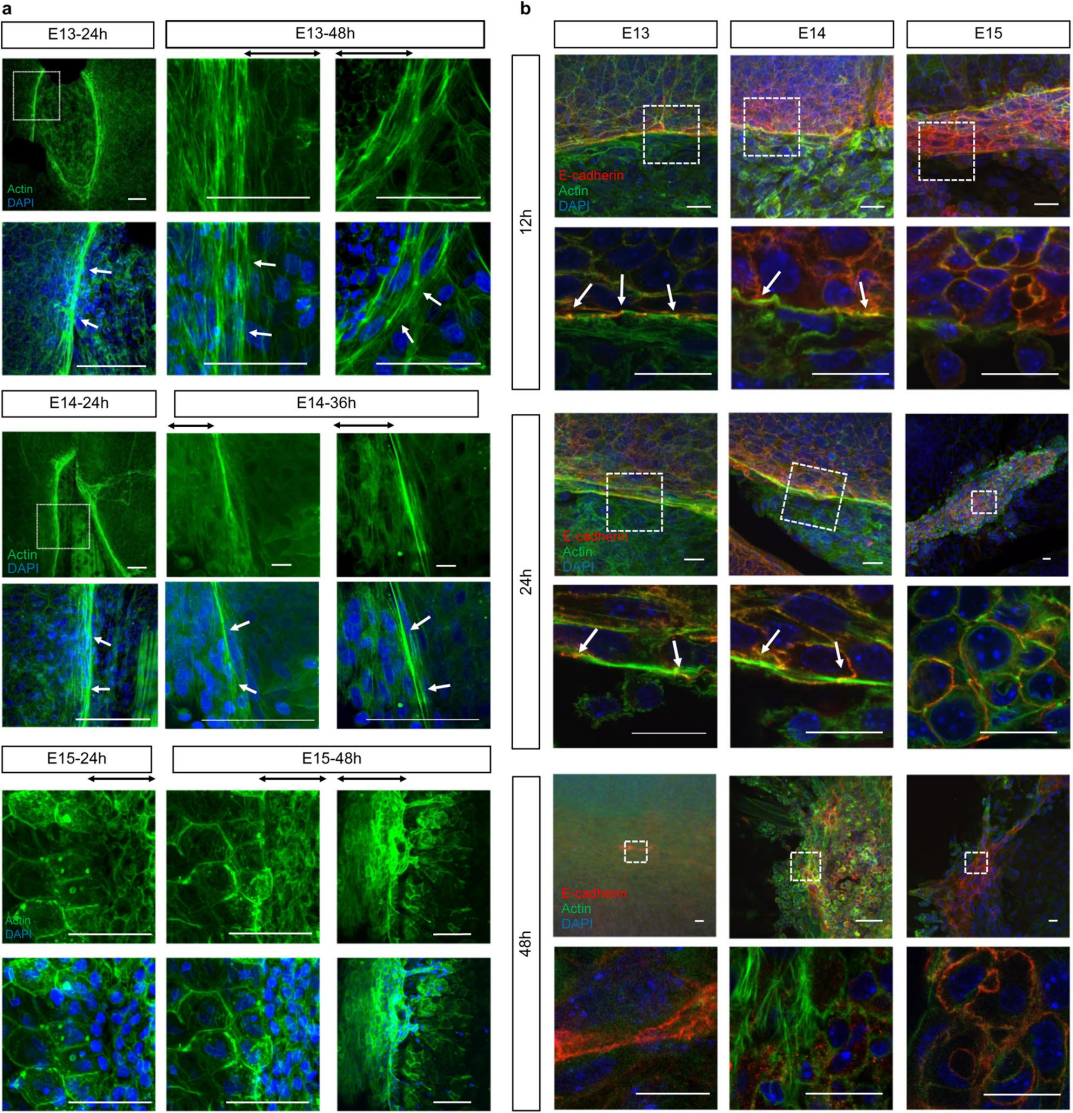

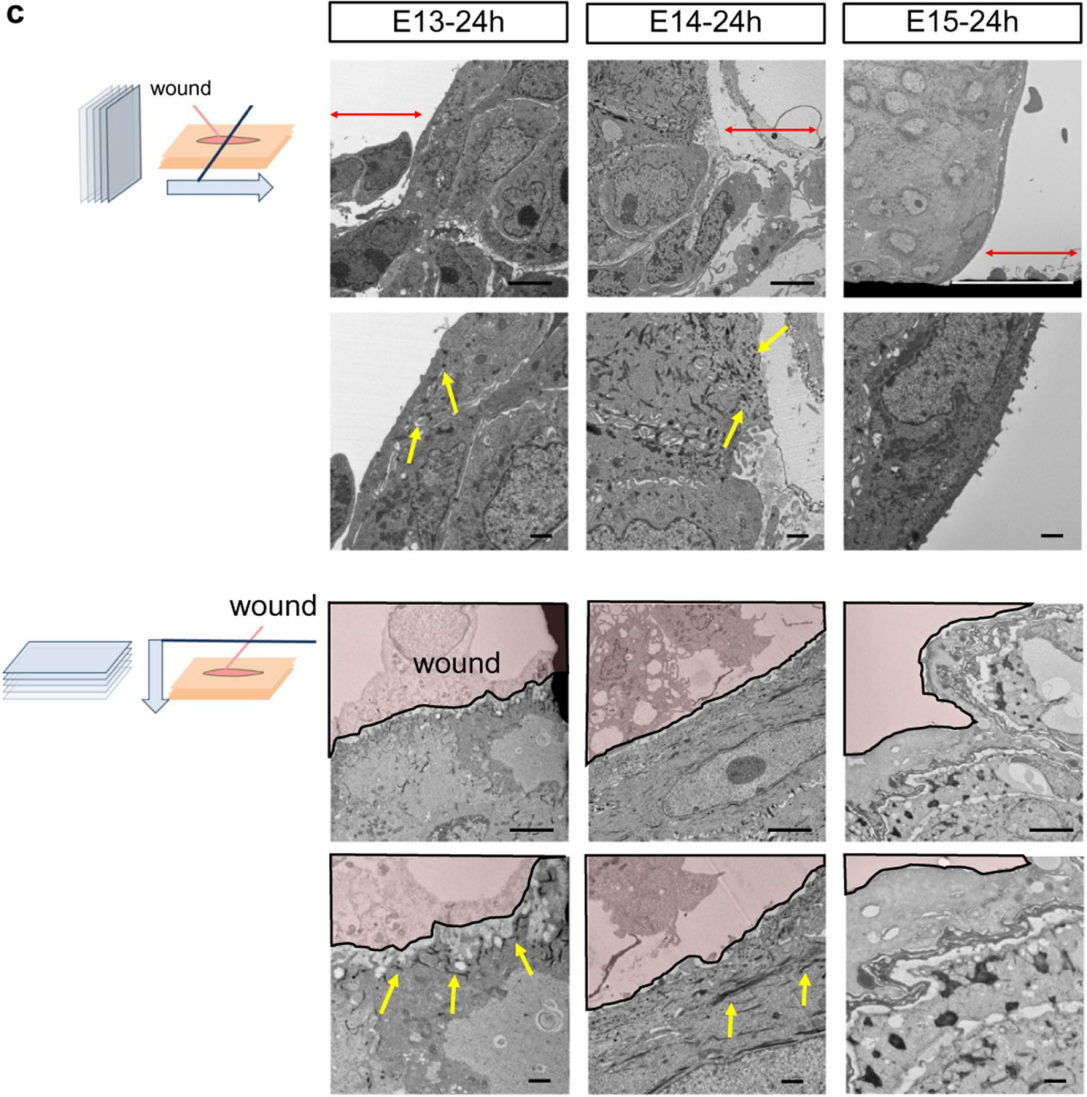
Formation of actin cables during mouse fetal wound healing. (**a**) Actin cable formation in E13–15 mouse fetuses. Actin cables were observed throughout the wound healing process at E13 and halfway through E14, but not at E15. Green: actin; blue: DAPI. (**b**) Actin cables and E-cadherin expression at 12, 24, and 48 h after wounding at E13–15. Anchoring by E-cadherin was observed at the wound site and during actin cable formation. Green: actin; red: E-cadherin; blue: DAPI. Scale bar = 20 µm. (**C**) TEM images of E13–15 wounds after 24 h. The wound is indicated by red arrows, and the actin cables are indicated by yellow arrows. Scale bar = 4 µm.

To further analyze the mechanism of complete epidermal regeneration, we analyzed the formation of actin cables in mouse fetal wounds. In wounds made at E13, actin cables formed throughout the entire process of wound closure. In E14 wounds, actin cables were observed up to 36 h after injury but disappeared afterward. In E15 wounds, actin cables did not form during early wound healing, and epidermal keratinocytes migration was observed (Fig. 4a).

To understand actin cable formation in more detail, we examined the relationship between actin filaments and E-cadherin, an intercellular adhesion molecule, as actin filaments must be conjugated with a molecule on the cell membrane to contract the wound. Twelve hours after injury, E-cadherin expression was enhanced at the wound edge in the area where the actin cable appeared and spread to neighboring cells in wounds made at E13 and E14. In addition, the actin cable was partially anchored to the cell membrane by E-cadherin at the wound edge. Twenty-four hours after injury, actin cables were enhanced at the wound edge in E13 and E14 wounds and merged with E-cadherin expression at the cell membrane and across neighboring cells. In contrast, in wounds made at E15, actin cables did not form during early wound healing. Forty-eight hours after injury, actin cables in E13 wounds contracted like a drawstring bag until wound closure. However, in E14 wounds, the actin cables disassembled. This pattern was similar to that observed in E15 wounds (Fig. 4b).

TEM examination of the wound margins 24 h after injury showed that, consistent with the histological findings, polymerized fibers were observed at the wound margins in E13 and E14 wounds, which were likely actin cables (Fig. 4c).

**Figure 5:**
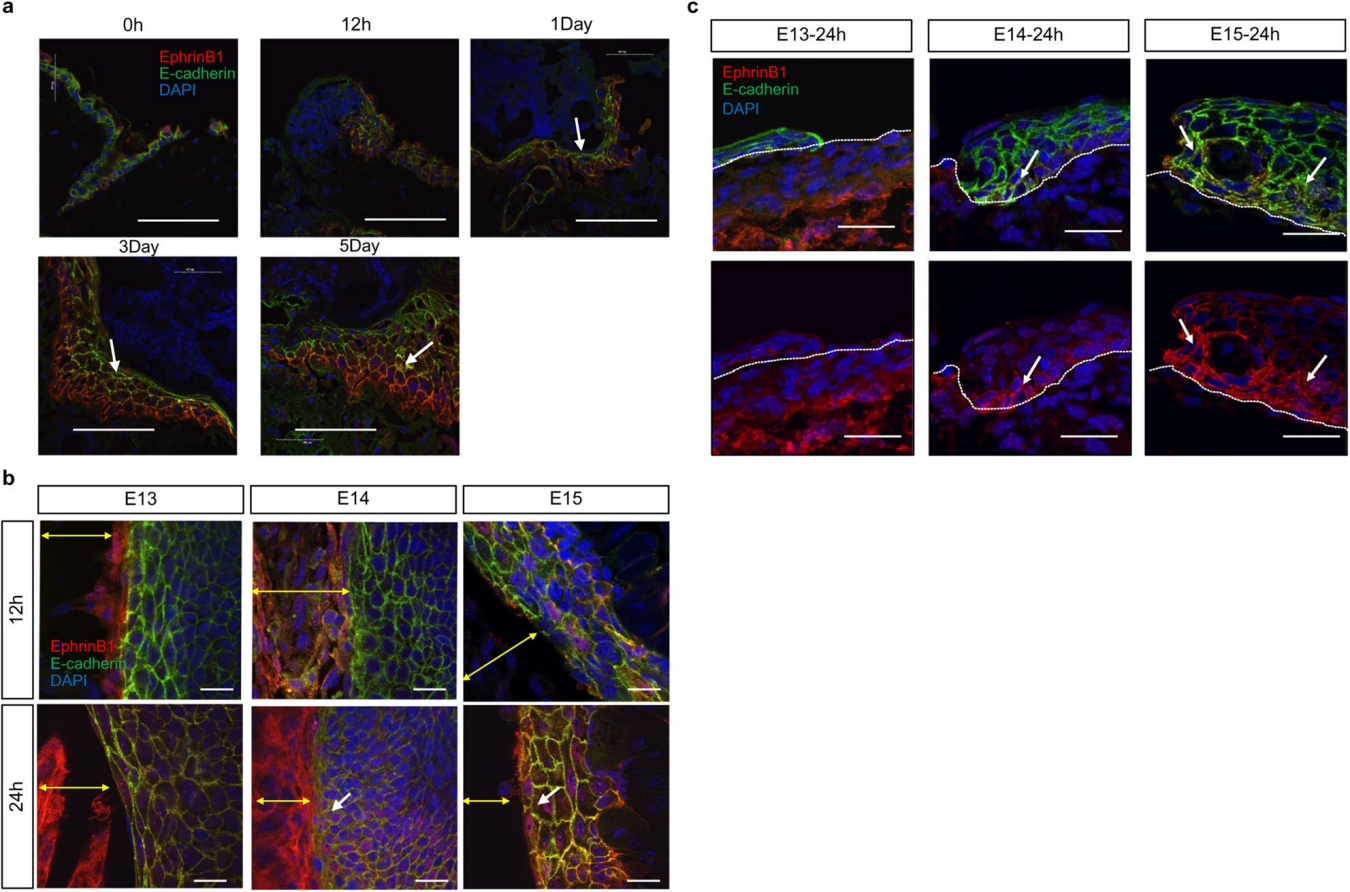
Relationship between ephrin-B1 and E-cadherin during wound healing. In E13 wounds, ephrin-B1 did not reduce E-cadherin levels. However, after E14, ephrin-B1 localized to the cell membrane and disassembled E-cadherin, suggesting a transition to cell migration-type wound healing. (**a**) Wounds in adult mice, Scale bar = 50 µm. (**b**) Whole-mount samples from fetal mice. Yellow arrow: wound. Scale bar = 20 µm. (**c**) Vertical section of the fetal wound. Scale bar = 30 µm, with arrows indicating areas of reduced E-cadherin expression. Dashed line: boundary between epidermis and dermis.

Martin et al. reported that ephrin-B1 expression at the wound margins in adult mice causes cell migration^20^. Therefore, we examined ephrin-B1 expression in the wound sites of adult and fetal mice. In adult mice, ephrin-B1 was enhanced on the cell membrane of the basal epidermis 24 h after creation. In addition, E-cadherin expression decreased in the area where ephrin-B1 expression was enhanced (Fig. 5a). In E13 fetuses, ephrin-B1 was expressed 24 h after wound creation, but no localization on the plasma membrane or disassembly of E-cadherin was observed. However, in E14 and E15 fetuses, ephrin-B1 was localized on the cell membrane of the epidermis on the dermal side, as observed in adult mice, and E-cadherin disassembly was observed (Fig. 5b,c). Therefore, cell migration in the epidermal limbus will likely occur after E14. These results suggest that at E13, when the skin completely regenerates, the epidermis shrinks like a drawstring due to actin cable contraction, and interactions between the epidermis and dermis are maintained. Thus, the skin regenerates through epidermal morphogenesis. In E14 wounds, the wounds closed from actin cable contraction and epidermal–dermal interactions until approximately 24 h after wound creation, but the actin cables disassembled after 24 h, and the epidermis migrated, suggesting that the epidermis contacted the fascia at the bottom of the wound before the dermis.

### Relationship between actin cable formation and AMPK activity

**Figure 6:**
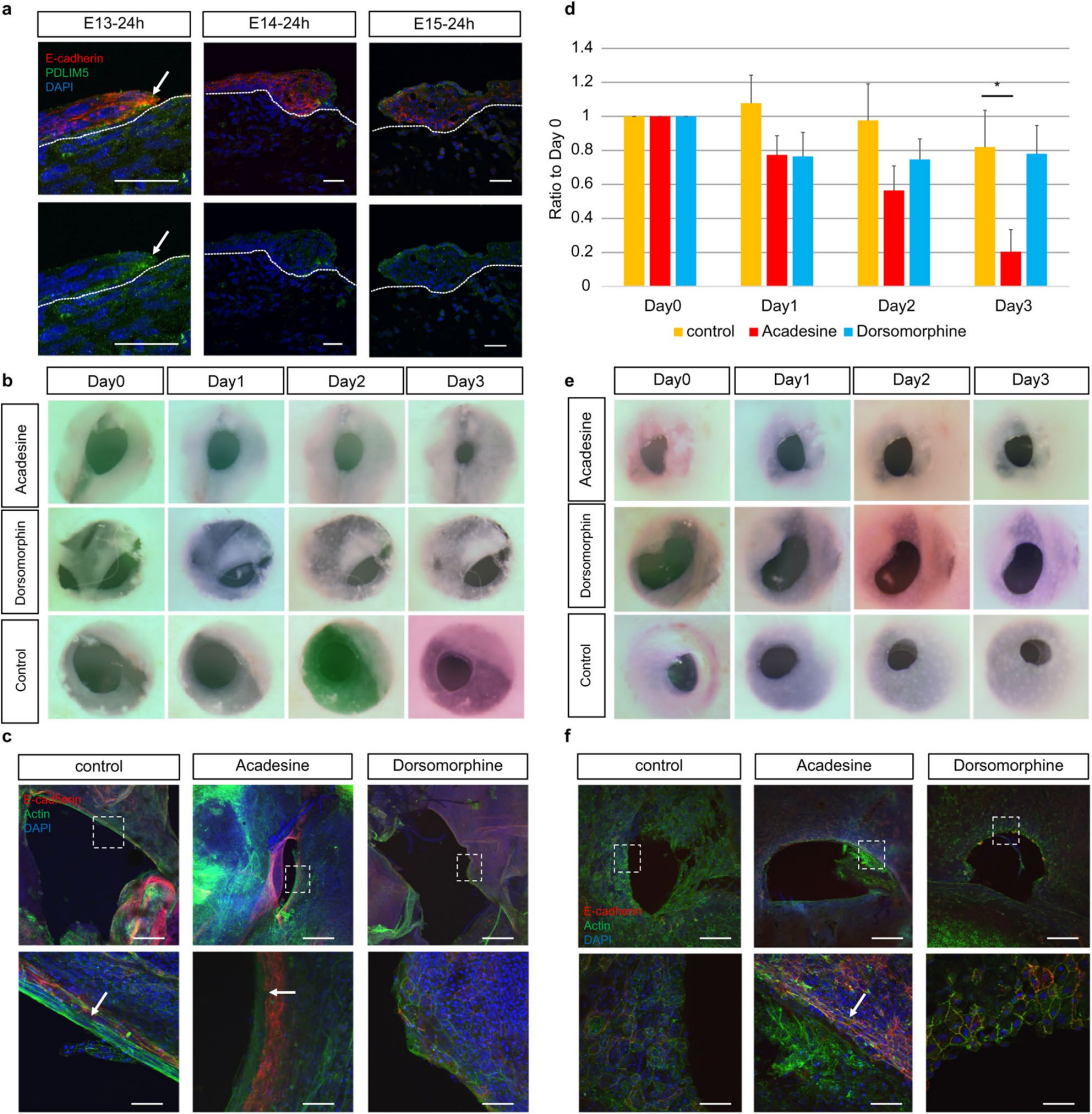

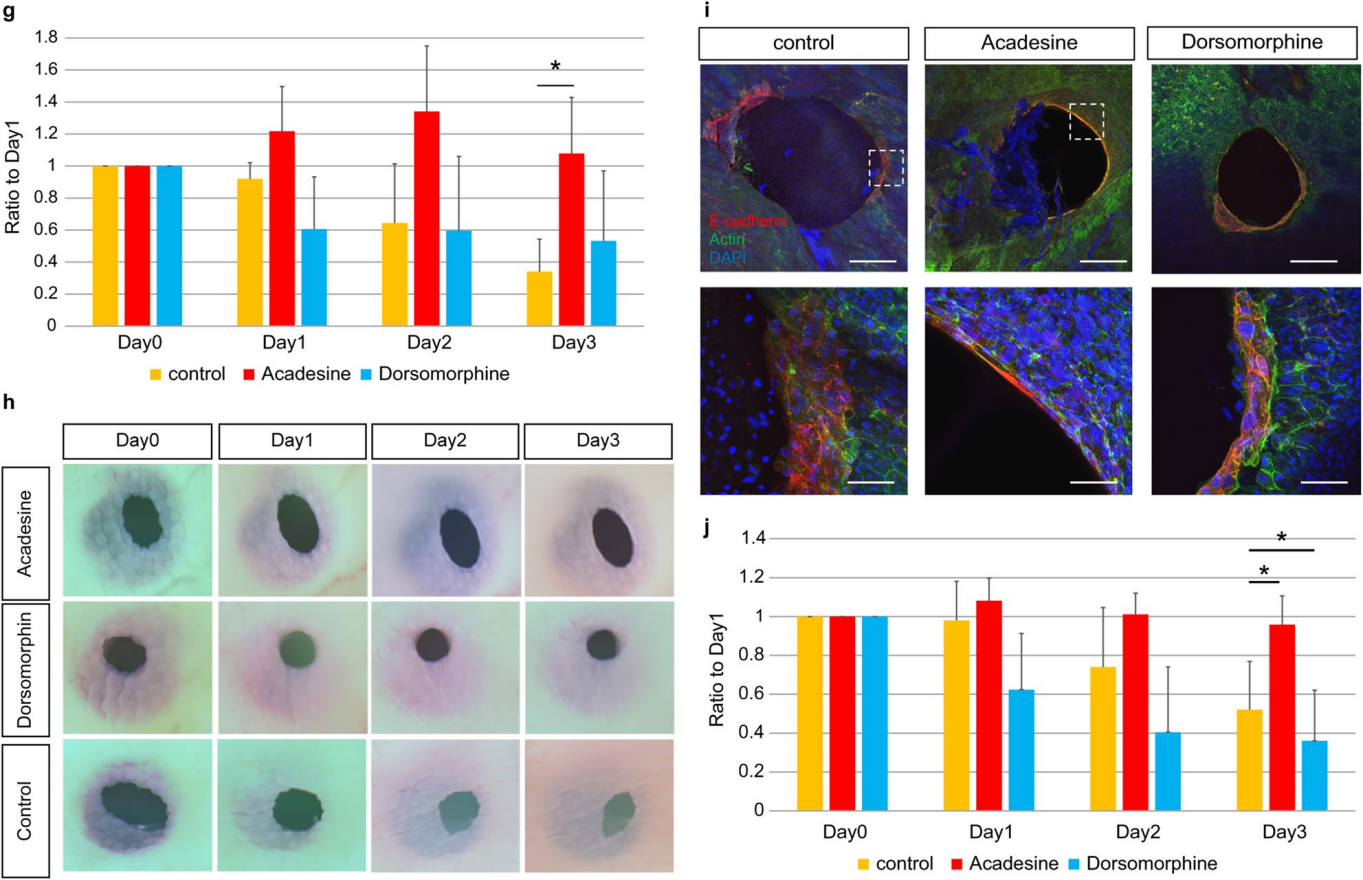
Effect of AMPK activity on wound healing ex vivo. (**a**) PDLIM expression during fetal wound healing. Scale bar = 30 µm. (**b**) Ex vivo regulation of AMPK activity and healing in E13 wounds. (**c**) Regulation of AMPK activity and actin cable formation in E13 wounds. (**d**) Wound area change due to the modulation of AMPK activity in E13 wounds. E: Ex vivo modulation of AMPK activity and healing in E14 wounds. (**ff**) Regulation of AMPK activity and actin cable formation in E14 wounds. (**g**) Change in wound area by modulating AMPK activity in E14 wounds. H: Ex vivo AMPK activity regulation and healing in E15 wounds. (**i**) Regulation of AMPK activity and actin cable formation in E15 wounds. (**j**) Changes in the wound area due to modulating AMPK activity in E15 wounds. *P < 0.05 (statistically significant).

Next, we examined whether actin cable formation is regulated by AMPK. PDLIM5 is present in actin cable structures such as stress fibers and focal adhesions, and Ser177 phosphorylation of PDLIM5 induced by AMPK inhibits cell migration, causing cells to switch from migration to stress fiber formation^23^. Therefore, we analyzed the relationship between PDLIM5 expression and the switch from the actin cable type to cell migration-type wound healing. We examined PDLIM5 expression in E13–15 mouse fetuses 24 h after injury and found that PDLIM5 expression was enhanced in the leading edge of the epidermis at the wound edge in E13 fetuses. In contrast, PDLIM5 expression was not enhanced in E14 and 15 wounds (Fig. 6a).

To investigate whether AMPK activity is involved in actin cable formation in epidermal tissues, E13–E15 skin was cultured ex vivo in medium containing acadesine (AMPK activation group) or dorsomorphin (AMPK inhibition group) for 3 days and morphological changes were evaluated. In E13 skin, we observed faster wound closure and smaller wounds at Day 3 in the AMPK-activated group than in the untreated group (P = 0.000004). The AMPK-activated group showed enhanced actin cable thickness compared to the untreated group. In E14 and E15 skin, the AMPK-activated group showed slower wound closure than the untreated group and significantly lower wound shrinkage on Day 3 (E14, P = 0.013; E15, P = 0.001), while the AMPK-suppressed group in the E15 group showed an increased rate of wound closure and a significantly higher rate of contraction (E15, P = 0.002). In the AMPK-suppressed group, as in the untreated group, actin was observed, which may be related to cell migration, but no actin cables were formed at the wound margin, whereas in the AMPK-activated group, actin cables were observed at the wound margin (Fig. 6b–j). These results suggest that AMPK activation facilitates skin regeneration using actin cables.

**Figure 7:**
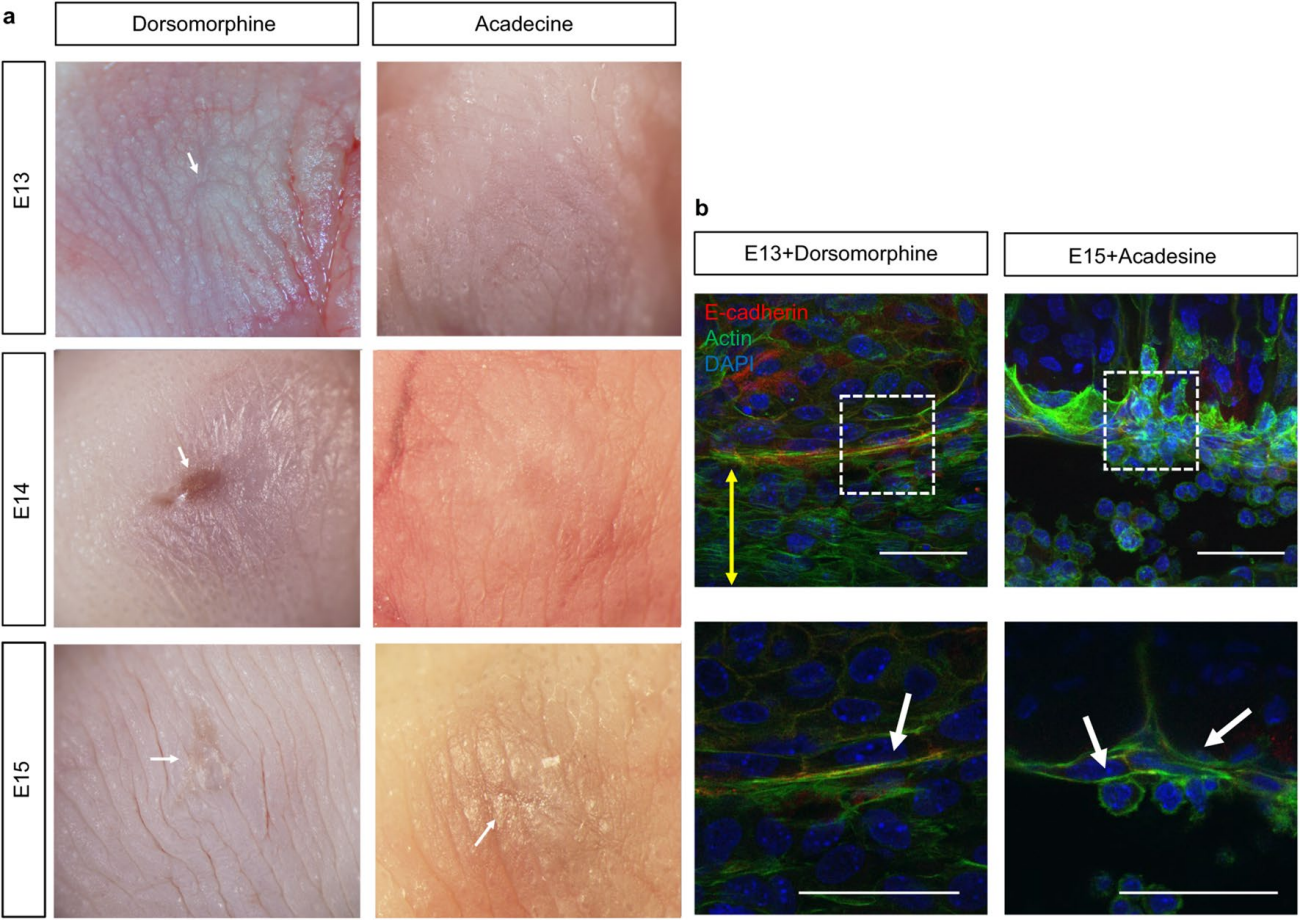
Effect of AMPK activity on wound healing in utero. (**a**) Effect of AMPK activity on wound healing 72 h after injury in E13, E14, and E15 wounds. Complete skin regeneration was observed in the AMPK activation group in E14 wounds. Arrow; visible mark. (**b**) In vivo regulation of AMPK activity and actin cable formation in E13 and E15 wounds. Yellow arrows indicate the wound area, and the white arrows indicate actin cables. Upper bar = 50 µm, lower bar = 20 µm.

We next examined the effect of modulating AMPK activity in utero on fetal wound healing. Previous results suggested that AMPK activation facilitated wound regeneration by actin cable formation. Seventy-two hours after wound creation at E13, a visible mark was observed in fetuses treated with dorsomorphin. Thus, AMPK activity is required for complete skin regeneration, including skin texture, as observed at E13 (Fig. 7a, Supplementary Fig. 2). Surprisingly, when acadesine was administered to E14 wounds, complete skin regeneration, including the skin texture, was observed after 72 h. At E15, wound healing was slower compared to the control, and a visible mark remained in the AMPK activation group. Immunohistological studies showed actin cable disassembly in the AMPK inhibition group in E13 wounds. However, we noted actin cable formation at the wound edge in the AMPK activation group in E15 wounds, confirming that the morphological changes in wound healing were due to the regulation of actin cable formation (Fig. 7b). Finally, no ephrin-B1 or PDLIM5 expression was found in the epidermis at the wound edge in AMPK-suppressed E13 wounds or AMPK-activated E15 wounds (Supplementary Fig. 3), which indicates that AMPK activity does not affect cell migration during wound healing.

## Discussion

Numerous studies have attempted to understand fetal wound healing mechanisms by comparing wound healing in fetal and adult animals. However, the two processes differ in many ways. Environmentally, the fetus is covered with sterile amniotic fluid that is rich in growth factors, whereas adult wounds are exposed to bacteria and other microorganisms that cause severe inflammation around the wound^25,26^. In addition, because the cells and tissues are at different stages of differentiation in fetal and adult animals, cross-reactions between different cells and molecules occur. Thus, it is not meaningful to simply compare fetal and adult wound healing to identify candidate molecules that cause scarless skin regeneration. Instead, it is important to focus on the transition between fetal wound healing without scarring and adult wound healing with scarring and to compare the two processes before and after this transition. These transitions, which were reported in other species, occur gradually rather than abruptly^12,27–29^. Most plastic surgeons are suspicious of the word "gradual" and generally divide wound healing into two phases—skin texture regeneration and dermal structure regeneration—to understand why scars develop. By focusing on skin texture regeneration and dermal regeneration, we were able to determine, for the first time, the transition point of wound healing from the fetal to the adult phenotype. We observed a rapid transition from a period of regeneration to a period of non-regeneration. Although it is unclear how the skin texture is formed, various lines of evidence suggest that it is triggered by epidermal–dermal interactions. By comparing skin morphology before and after this transition, we found that during wound healing at E13, the wound closed via an epidermal–dermal interaction. Conversely, an epidermal–fascial interaction occurred during wound closure at E14. Previous reports showed that epidermal development and postnatal hair follicle formation are regulated by reciprocal beneficial interactions between the epithelium (epidermis) and mesenchyme (dermis), as shown in co-culture experiments of epidermal and dermal components from embryonic skin^30^. Epidermal cells receive beneficial signals from the underlying dermis, and mesenchymal cells require epidermal signals to form dermal papillae^31,32^.

We hypothesized that similar to epidermal morphogenesis, dermal mesenchymal cells regenerate skin texture by interacting with epidermal cells, whereas fascial mesenchymal cells do not. To clarify this hypothesis, we previously found that when an E16 fetal-derived epidermal sheet was transplanted directly into the fascial layer of another E16 fetus, the skin texture was lost in the absence of dermal mesenchymal cells^32^. By transplanting E17-derived fascial mesenchymal cells into the E13 dermis, E13 wounds lost the ability to regenerate epidermal patterning, but the E17 dermal mesenchymal cells did not destroy this ability^33^. These results indicate that dermal mesenchymal cells play an important role in skin texture regeneration.

Regarding dermal regeneration, previous reports showed that adult wound scars are composed of fascial mesenchymal cells^34–36^. Our previous report showed that during fetal wound healing during the late embryonic period, fascial mesenchymal cells construct scar tissue, as in adult animals, but during wound healing that occurs when the dermis regenerates, the dermal mesenchymal cells promote dermal regeneration^33^.

We also found for the first time that a switch from actin cable formation to cell migration occurs during complete skin regeneration, including epidermal texture regeneration. Actin cables are involved in epithelial regeneration in other animal species^37,38^. In our study, actin cables were observed in E13 wounds and were formed by E-cadherin-bundled actin cables that extend horizontally against the wound edge. In addition, PDLIM5 expression was enhanced in the apical part of the epidermis at the wound edge, and cell migration was inhibited. In contrast, no PDLIM5 expression was observed in E15 wounds, and cell migration was accompanied by enhanced ephrin-B1 expression and reduced E-cadherin expression. Previous reports showed that during wound healing in adult mice, ephrin-B1 and its receptor, ephrin-B2, are enhanced in the epidermis at the wound margins, thereby loosening E-cadherin-induced cell adhesion and causing cell migration^20^. The gradual change in actin cable loss from E13 to E15 observed in this study coincides with the time of the switch in epidermal–dermal interactions.

We also altered the activity of AMPK, which is involved in actin cable formation, to control scarring. AMPK is a heterotrimeric serine/threonine kinase consisting of a catalytic α-subunit and regulatory β-and γ-subunits, all of which are highly conserved in eukaryotes^39^. AMPK is activated under stress conditions via αThr172 phosphorylation to regulate cellular and systemic energy homeostasis^40^. Enhanced AMPK activity inhibits cell migration and may prevent atherosclerosis and cancer metastasis. However, the underlying mechanism is largely unknown^41^. However, the ability of AMPK to regulate cell migration suggests that this molecule is associated with wound healing. For example, resveratrol, an AMPK activator, promotes surgical wound healing, while adenine, which also activates AMPK, inhibits scar formation^24^. Additionally, AMPK regulates cell migration by controlling microtubule and actin cable behavior. PDLIM5, which was recently identified as an AMPK substrate, inhibits cell migration by suppressing the Ras-related C3 botulinum toxin substrate 1 (Rac1)-actin-related protein (Arp) 2/3 signaling pathway^42^.

While AMPK inhibited cell migration at E14 and E15, it promoted actin cable formation, which completely regenerated the skin in E14 wounds. In contrast, suppressing AMPK in E13 wounds resulted in scar formation, suggesting that AMPK activation may result in embryonic-type wound healing. However, we could not confirm whether ephrin-B1 expression was enhanced by AMPK suppression or whether PDLIM5 expression was enhanced by AMPK activation. In adult animals, AMPK regulation alone did not lead to complete skin regeneration, but in E13 skin, AMPK inhibition prevented skin texture regeneration, which would normally be regenerated. In E14 wounds, AMPK activation promoted skin texture regeneration. Thus, factors other than AMPK activity may be involved in wound healing in adult animals. However, further investigation is necessary.

In conclusion, using our original mouse fetal wound healing model, we have shown that complete skin regeneration in mouse fetuses occurs between E13 and E14, and dermal regeneration occurs between E16 and E17 when it switches to an adult "repair" type of healing. In addition, complete skin regeneration, including texture, was achieved by controlling AMPK activity. Although further studies on other factors that inhibit cell migration and promote actin cable formation are needed, the results of this study may contribute to the development of scarless wound healing.

## Materials and methods

The research protocol was reviewed and approved by the Institutional Animal Care and Use Committee of Keio University School of Medicine (approval number: 20170914). All experiments were conducted in accordance with the institutional guidelines for animal experiments at Keio University. This study is reported in accordance with the Animal Research: Reporting of In Vivo Experiments (ARRIVE) guidelines.

### Fetal wounding procedure

ICR mice were used in this experiment. All mice were obtained from the Sankyo Lab Service Co. Vaginal plugs were checked twice daily. When a plug was observed, the fetus was designated as E0. The fetuses were wounded at E13, E14, E15, E16, and E17. Surgery was performed on five pregnant mice at each time point. Pregnant mice were anesthetized with isoflurane, and the abdominal wall was incised to expose the uterus. Using an operating microscope, an incision was made in the myometrium and the amnion/yolk sac. Then, a full-layer incision wound approximately 2 mm long was made in the lateral thorax of the fetus using surgical micro-scissors. In the skin excision model, a 1.0 mm diameter skin was excised from the lateral chest using a self-made motorized skin biopsy punch. At E13 and E14, after wounding, the amnion and yolk sac were sutured with 9-0 nylon, but the myometrium was left open and unsutured. In other words, the fetus was returned to the abdominal cavity with the amnion and yolk sac covered, but not the myometrium, and the abdomen was closed. On E15, E16, and E17, after the fetal wound was created, the myometrium was sutured with 9-0 nylon, the uterus was returned to the abdominal cavity, and the abdomen was closed. Then, 1 µg/g body weight of the uterine relaxant ritodrine hydrochloride (FUJIFILM Wako Pure Chemical Co., Ltd.) was administered intraperitoneally immediately before the closure of both wounds. Then, the peritoneum and skin were sutured with 5-0 nylon thread. The surgical methods used for each embryonic period were developed empirically. When fetuses were retrieved 36, 48, or 72 h after surgery, the wound was labeled with 0.25% 1,1′-Dioctadecy-3,3,3′,3′-tetramethylindocarbocyanine perchlorate (DiI) dissolved in 1% ethanol in phosphate-buffered saline (PBS) to mark the wound area and for visualization. The maternal mice were euthanized by cervical dislocation, and the fetuses were harvested 0, 6, 9, 12, 15, 18, 24, 36, 48, and 72 h after wounding. For each time point, wounds were created in at least four fetuses. DiI fluorescence in the wounds was examined using a stereomicroscope (SZX16, Olympus Co., Ltd., Tokyo, Japan) and an ultra-high-precision digital microscope VHX-8000 (Keyence Co., Ltd., Osaka, Japan). Morphological images of the wounds after 72 h were imported into ImageJ, the wound area was measured, and the area ratio was calculated. The skin of the fetal wound area was collected and fixed in 4% paraformaldehyde (PFA) for 24 h. The fixed tissue was embedded in paraffin and stained. For histology, hematoxylin and eosin (H&E) staining was performed using 7-µm sections. For immunostaining, the tissue was immersed in 20% sucrose/PBS after fixation, frozen, embedded in OCT compound (Sakura Finetek Japan Co., Ltd, Tokyo, Japan), and sliced at 7 µm.

### Scanning electron microscopy (SEM)

Fetal wounds were cut into 1 mm squares for SEM observation, pre-fixed in 4% paraformaldehyde/2% glutaraldehyde/0.05 M cacodylate (pH 7.4) for 2 h at room temperature (20–23 °C), and washed five times with 0.05 M cacodylate for 5 min. The tissue was post-fixed in 1% osmium tetroxide/0.05 M cacodylate for 2 h at room temperature. The tissues were washed three times with distilled water and dehydrated with 25, 50, 75, 90, and 100% ethanol for 30 min each. Then, the tissue was immersed in a mixture of equal parts 100% ethanol and isoamyl acetate for 30 min. The tissue was soaked in 100% isoamyl acetate twice for 15 min at room temperature, and the samples were dried using a critical point drying apparatus (SYGLCP-81, Sanyuu Gijutsu, Co., Ltd., Tokyo, Japan). The samples were mounted on a sample stand and observed under a scanning electron microscope (JSM 7500F, JEOL, Ltd., Tokyo, Japan).

### Transmission electron microscopy (TEM)

Fetal wounds were cut into 1 mm squares for SEM observation. The sections were pre-fixed in 2.5% glutaraldehyde-0.1 M phosphate buffer (pH 7.4) for 2 h at 4 °C and washed three times with 0.2 M phosphate buffer for 10 min. The tissues were then post-fixed in 1% osmium tetroxide-0.1 M phosphate buffer (pH 7.4) for 2 h at 4 °C, washed three times with 0.2 M phosphate buffer for 10 min, and dehydrated in 50, 70, 80, 90, and 100% ethanol for 30 min each. Next, the samples were placed in *N*-butyl glycidyl ether (QY-1) for 10 min twice, followed by embedding in an equal volume mixture of QY-1 and epoxy resin overnight at room temperature. Samples were then sliced at a thickness of 60 µm using an ultramicrotome diamond knife, stained with 3% platinum blue, and observed with a transmission electron microscope Tecnai F20 (Koninklijke Philips NV, Inc., Amsterdam, The Netherlands).

### Adult mouse wounding procedure

Ten-week-old male ICR mice were anesthetized with isoflurane, and two full-layer wounds (approximately 1 cm in diameter) per mouse were created on the bilateral thorax using a No. 11 scalpel on the shaved back skin. This surgery was performed on five adult mice. The mice were euthanized by cervical dislocation 0, 12, 24, 72, and 120 h after wounding. Then, the wounds were excised, collected, and fixed in 4% PFA overnight. The samples were immersed in 20% sucrose/PBS, embedded in OCT compound, and sliced at a thickness of 10 µm.

### Immunohistochemistry

Tissue samples on a slide were washed three times with 0.2% Triton X-100 in PBS (PBST) for 10 min. Cells were washed once with PBS for 30 s and blocked with 3% bovine serum albumin (BSA)/PBS solution for 1 h at room temperature. For actin staining, the cells were incubated with Acti-stain 488 phalloidin (PHDG1-A; Cytoskeleton, Inc., Denver, CO, USA; 1:140) overnight at 4 °C. After washing three times with PBST, the nuclei were labeled with Cellstain DAPI Solution (FUJIFILM Wako Pure Chemical Co., Ltd.; 1:500) and mounted on glass slides with ProLong Gold (Invitrogen).

For immunostaining against other antigens, after blocking, primary antibodies were diluted in a blocking solution (3% BSA/PBS), and the tissue was incubated in antibody solution overnight at 4 °C using the following antibodies: rat anti-E-cadherin (SAB4200684; Sigma-Aldrich, St. Louis, MO, USA; 1:200), goat anti-ephrin-B1 (AF496; R&D Systems, Minneapolis, MN, USA; 1:200), and rabbit anti-Pdlim5 (10530-1-AP; Proteintech, Rosemont, IL, USA; 1:100). Then, the samples were washed three times with PBST and incubated with Hoechst 33342 (1:500) and the following secondary antibodies for 1 h: AlexaFluor (Invitrogen, Waltham, MA, USA. ab150158; goat anti-rat IgG Alexa Fluor 555 or ab150078; goat anti-rabbit IgG Alexa Fluor 555; 1:200) or Cy3 donkey anti-goat IgG (H + L) antibody (2307351; Jackson ImmunoResearch, West Grove, PA, USA; 1:200). The cells were washed twice with PBST, once with PBS, and mounted on glass slides using ProLong Gold (Invitrogen). All slides were observed under a confocal laser scanning microscope (FLUO-VIEW FV3000; Olympus, Co., Ltd.).

### Ex vivo skin tissue culture

Skin from E13 and E15 mouse fetuses was removed in full layers and pasted onto filter paper with a 2-mm hole in the center. Skin was collected from five fetuses from each day. Then, the skin was punctured with a 1-mm skin biopsy punch so that the centers of the holes coincided. The skin was then cultured with filter paper. Dulbecco's modified Eagle's medium (DMEM) containing 10% FBS and 1% penicillin/streptomycin (FUJIFILM Wako Pure Chemical Co., Ltd., Osaka, Japan) was used as the standard culture medium. The cells were cultured for 3 days in a culture medium containing the AMPK activator acadesine (Sigma-Aldrich; 2 mM) or the inhibitor dorsomorphin (Tokyo Chemical Industry Co., Ltd, Tokyo, Japan; 2 µL). Images were imported into ImageJ, and the wound area was evaluated by measuring the area around the edges of the hole using polygonal sections. The area on day 0 was set to 1, and the ratio of the area of the wound at each time point was calculated.

### Regulation of AMPK activity in vivo

E13 and E15 fetuses underwent wound surgery and received 100 µL acadesine (20 mM) or dorsomorphin (20 µM) in the amniotic fluid. At each time point, three maternal mice and four fetuses per maternal mouse, i.e., 12 fetuses for each, were subjected to this treatment. Reagents were administered during the surgery for E13 and E15 fetuses and the day after surgery for E14 fetuses. PBS:DMSO (500:1) was used as the control. Red fluorescent dye (DiI) was used to stain the wounds, which were imaged using the 4× objective lens of a BZ-X710 all-in-one fluorescence microscope (Keyence, Co., Ltd., Tokyo, Japan). Seventy-two images were captured and concatenated to obtain a wide-field image. The skin texture of the area corresponding to the wound was photographed using an MJ-MS302 digital USB microscope (Sato Shoji Co., Ltd., Kanagawa, Japan).

### Statistical analysis

Box-and-whisker plots show the entire population. Other data are expressed as mean ± standard error of mean. Statistically significant differences between the two groups were determined using Statistica software version 9.0 (StatSoft, Tulsa, OK). Differences were considered significant at P < 0.05. An unpaired Student's *t*-test was used to analyze differences between the two groups. One-way analysis of variance (ANOVA) and Tukey's post hoc tests were used to compare differences between three or more groups. A p-value < 0.05 was considered significant.

## Supplementary Information

Supplementary Figure 1.

Supplementary Figure 2.

Supplementary Figure 3.

Supplementary Legends.

## Data Availability

The data that support the findings of this study are available from the corresponding author, K.K., upon reasonable request.
