# Supplementary figures and images for: Actin cable formation and epidermis–dermis positional relationship during complete skin regeneration
Source: Sci Rep. 2022 Sep 23;12:15913. doi: 10.1038/s41598-022-18175-y (PMC9508246; doi:10.1038/s41598-022-18175-y)

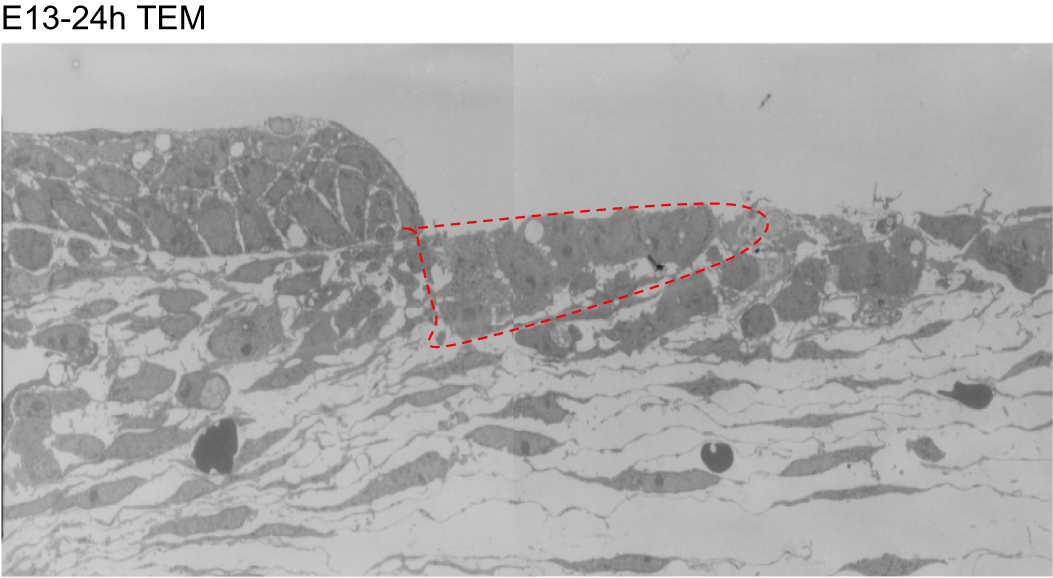

Supplement: Supplementary file 1 — Supplementary Figure 1. [file 41598_2022_18175_MOESM1_ESM.tif]

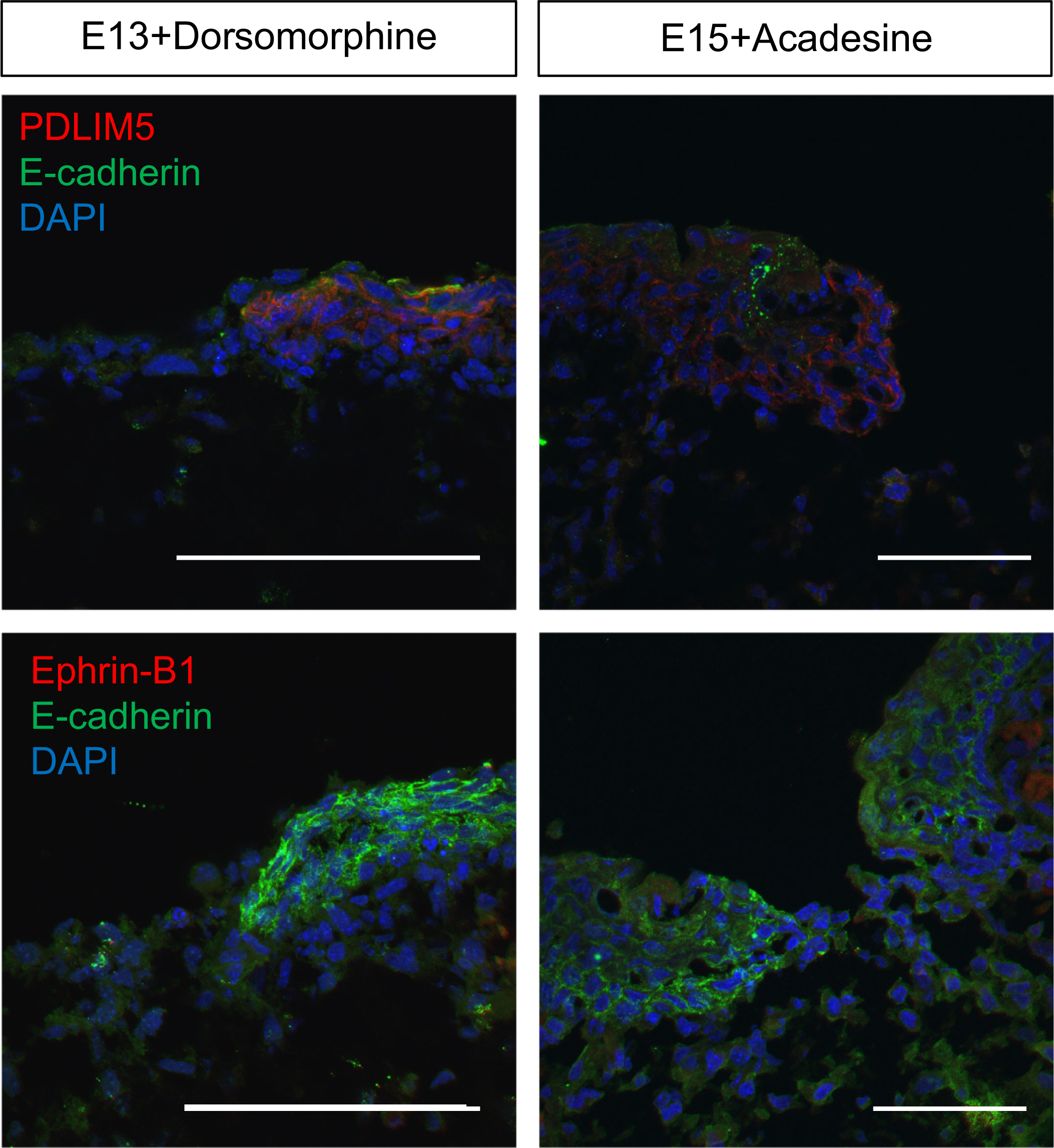

Supplement: Supplementary file 2 — Supplementary Figure 2. [file 41598_2022_18175_MOESM2_ESM.tif]

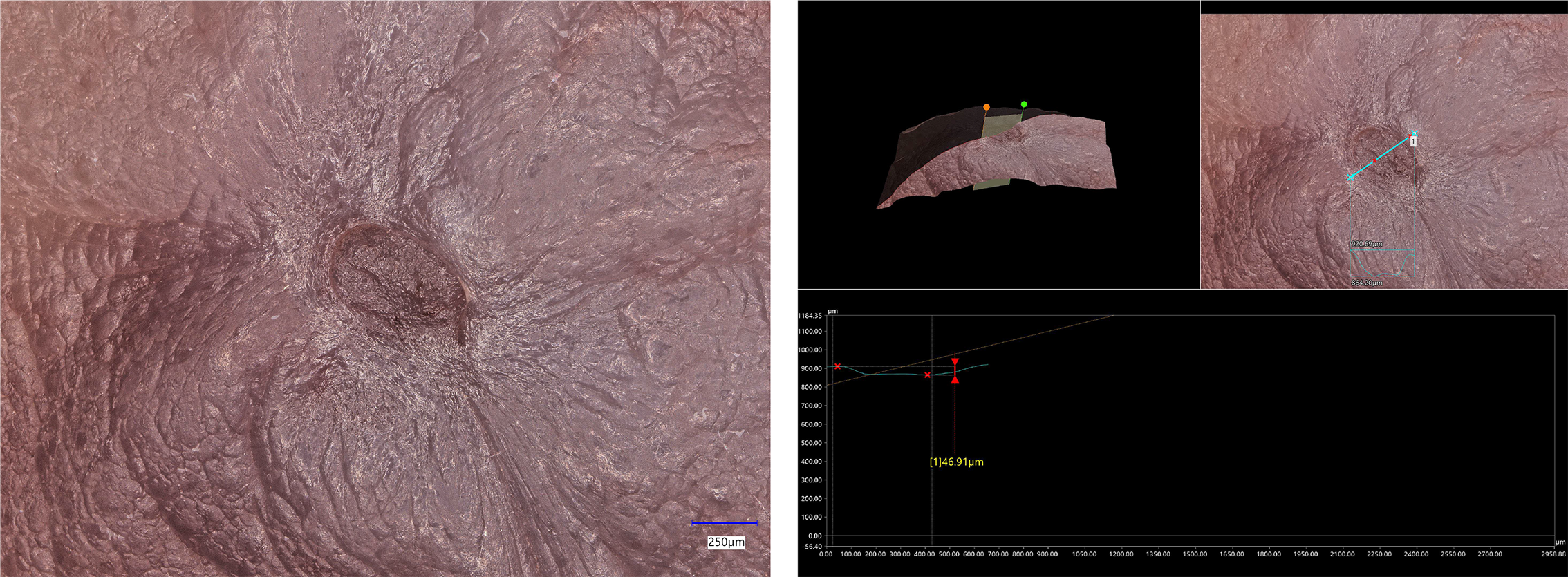

Supplement: Supplementary file 3 — Supplementary Figure 3. [file 41598_2022_18175_MOESM3_ESM.tif]
