# Supplementary material for: Actin cable formation and epidermis–dermis positional relationship during complete skin regeneration
Source: Sci Rep. 2022 Sep 23;12:15913. doi: 10.1038/s41598-022-18175-y (PMC9508246; doi:10.1038/s41598-022-18175-y)
Supplement: Supplementary file 4 — Supplementary Legends. [file 41598_2022_18175_MOESM4_ESM.docx]

**Supplementary Figure Legends**

Supplementary Fig. 1 Transmission electron microscopy image of an E13 wound at 24 h. Chromatin-rich cells, including dermal cells, were observed at the tip of the wound (red line). Scale bar=200 µm.

Supplementary Fig. 2 Inhibition of skin regeneration by dorsomorphine in E13 wounds. A visible mark remained, and a depressed scar was observed in the surrounding area.

Supplementary Fig. 3 *In vivo* AMPK activity and PDLIM5 and ephrin-B1 expression.

Scale bar=60 µm.
